# Evaluation of daytime sleepiness and insomnia symptoms in OSA patients with a characterization of symptom-defined phenotypes and their involvement in depression comorbidity

**DOI:** 10.1192/j.eurpsy.2024.806

**Published:** 2024-08-27

**Authors:** A. Gabryelska, S. Turkiewicz, P. Bialasiewicz, F. Grzybowski, D. Strzelecki, M. Sochal

**Affiliations:** ^1^Department of Sleep Medicine and Metabolic Disorders; ^2^Department of Affective and Psychotic Disorders, Medical University of Lodz, Lodz, Poland

## Abstract

**Introduction:**

Recent studies have emphasized the importance of clinical manifestations, such as insomnia and sleepiness, in defining phenotypes of obstructive sleep apnea (OSA), shifting from a focus on OSA severity and sleep structure.

**Objectives:**

The study aimed to characterize insomnia and sleepiness associated with OSA phenotypes and assess their involvement in depression symptoms (DS) in OSA.

**Methods:**

A total of 181 participants undergoing polysomnography (PSG) were asked to fill out questionnaires, including Epworth Sleepiness Scale (ESS), Insomnia Severity Index (ISI), Pittsburgh Sleep Quality Index (PSQI), and Back Depression Index (BDI). They were categorized into phenotypes: insomnia-sleepiness (I+S; ESS≥11; ISI≥15; n=20), sleepiness (S; ESS≥11; ISI<15; n=22), insomnia (I; ESS<11; ISI≥15) and asymptomatic (A; ESS<11; ISI<15; n=55).

**Results:**

A linear regression model for BDI score (R^2^=0.357, p<0.001) included ISI score and subjective to objective sleep latency ratio. ISI score was a predictive factor for mild and moderate DS (OR=1.226, p<0.001 and OR=1.392, p=0.002, respectively). I and I+S phenotypes are characterized by higher BDI scores (p<0.001 and p=0.015), longer subjective sleep latency (p=0.008 and p=0.041), and shorter subjective total sleep time (TST; p=0.049 and p=0.006), compared to A. Furthermore, the I and I+S groups had shorter subjective TST than S (p=0.028 and p=0.047). I and I+S had higher BDI scores than A (p<0.001 and p=0.015, respectively) and S (p<0.001 and p=0.017, respectively). I phenotype was associated with the risk of mild and moderate DS (OR=5.614, p<0.001 and OR=9.550, p=0.008 respectively). Moreover, the I+S phenotype presented an even greater risk for mild DS (OR=10.286, p<0.001).

**Conclusions:**

The study suggests that using clinical features for OSA phenotyping holds promise for finding OSA individuals with increased risk for the occurrence of DS.

**Disclosure of Interest:**

None Declared

